# Saudi Familial Hypercholesterolemia Patients With Rare *LDLR* Stop Gain Variant Showed Variable Clinical Phenotype and Resistance to Multiple Drug Regimen

**DOI:** 10.3389/fmed.2021.694668

**Published:** 2021-06-25

**Authors:** Zuhier Ahmed Awan, Omran M. Rashidi, Bandar Ali Al-Shehri, Kaiser Jamil, Ramu Elango, Jumana Y. Al-Aama, Robert A. Hegele, Babajan Banaganapalli, Noor A. Shaik

**Affiliations:** ^1^Department of Clinical Biochemistry, Faculty of Medicine, King Abdulaziz University, Jeddah, Saudi Arabia; ^2^Department of Genetics, Al Borg Medical Laboratories, Jeddah, Saudi Arabia; ^3^Department of Genetic Medicine, Faculty of Medicine, King Abdulaziz University, Jeddah, Saudi Arabia; ^4^Princess Al-Jawhara Center of Excellence in Research of Hereditary Disorders, King Abdulaziz University, Jeddah, Saudi Arabia; ^5^Department of Genetics, Bhagwan Mahavir Medical Research Center (BMMRC), Hyderabad, India; ^6^Departments of Medicine and Biochemistry, Schulich School of Medicine and Dentistry, Robarts Research Institute, Western University, London, ON, Canada

**Keywords:** familial hypercholesterolemia, genetic diagnosis, monogenic diseases, consanguineous populations, LDLR pathogenic mutations

## Abstract

Familial hypercholesterolemia (FH), a well-known lipid disease caused by inherited genetic defects in cholesterol uptake and metabolism is underdiagnosed in many countries including Saudi Arabia. The present study aims to identify the molecular basis of severe clinical manifestations of FH patients from unrelated Saudi consanguineous families. Two Saudi families with multiple FH patients fulfilling the combined FH diagnostic criteria of Simon Broome Register, and the Dutch Lipid Clinic Network (DLCN) were recruited. LipidSeq, a targeted resequencing panel for monogenic dyslipidemias, was used to identify causative pathogenic mutation in these two families and in 92 unrelated FH cases. Twelve FH patients from two unrelated families were sharing a very rare, pathogenic and founder LDLR stop gain mutation i.e., c.2027delG (p.Gly676Alafs^*^33) in both the homozygous or heterozygous states, but not in unrelated patients. Based on the variant zygosity, a marked phenotypic heterogeneity in terms of LDL-C levels, clinical presentations and resistance to anti-lipid treatment regimen (ACE inhibitors, β-blockers, ezetimibe, statins) of the FH patients was observed. This loss-of-function mutation is predicted to alter the free energy dynamics of the transcribed RNA, leading to its instability. Protein structural mapping has predicted that this non-sense mutation eliminates key functional domains in LDLR, which are essential for the receptor recycling and LDL particle binding. In conclusion, by combining genetics and structural bioinformatics approaches, this study identified and characterized a very rare FH causative LDLR pathogenic variant determining both clinical presentation and resistance to anti-lipid drug treatment.

## Introduction

FH (OMIM 143890) is a relatively common metabolic disease in which patients demonstrate life-long elevation of plasma low-density lipoprotein (LDL) cholesterol (1). If left untreated, modified LDL particles enter arterial wall macrophages contributing to plaque formation, particularly within coronary arteries leading to premature development of coronary heart disease (CHD) (2). Cholesterol-laden macrophages lead to formation of not only atheromatous plaques, but also extensor tendon xanthomas (e.g., Achilles and fingers), xanthelasmata (yellow deposit underneath the skin of upper and lower eyelids), and arcus cornealis (cholesterol ring accumulating at the edge of the cornea) (3). Since FH is asymptomatic in the initial stages, most FH patients do not realize their illness until the onset of symptomatic atherosclerotic cardiovascular disease in their forties or fifties, which is sometimes fatal (4). The overall prevalence of FH in the Gulf region is estimated to be ~ 0.43% (1/232), however, in Saudi Arabia, prevalence of FH is not yet established due to the dearth of local FH clinical registries, epidemiological studies, and population genetic screening programs (5, 6). Diagnosis rates of FH are quite high among individuals who have a positive family history of premature CHD or hypercholesterolemia (7).

FH is caused by defective hepatic uptake of LDL receptor (R) mediated LDL-C particle degradation processes. About 30–60% of clinically diagnosed FH patients have a single copy of a pathogenic mutation (8). Majority of the clinically diagnosed FH patients (~80%) have a mono-allelic loss-of-function (LoF) variant in *LDLR* gene, while the rest are LoF variants in the receptor-binding domain of the *APOB* gene or a gain-of-function (GoF) variant in the *PCSK9* gene. A very small proportion of FH have biallelic LoF mutations in *LDLRAP1* which normally assists in LDL receptor internalization by liver cells. However, ~30–70% of clinically diagnosed FH patients are negative for *LDLR, APOB, or PCSK9* pathogenic mutations. Few have very rare LoF mutations in secondary FH genes, including *ABCG5, ABCG8, LIPA*, or *APOE*, while many other patients with hypercholesterolemia carry several common genetic variants (also called single nucleotide polymorphisms), which collectively act to influence the serum LDL-C concentration (9).

FH typically shows either autosomal dominant (HeFH; heterozygous FH) or autosomal recessive (HoFH; homozygous FH) mode of inheritance based on one or two copies of pathogenic variants in *LDLR, APOB or PCSK9* genes (10, 11). In most of the studied populations, HoFH affects 1 in 160,000–300,000 individuals, while the HeFH affects out 1 in 250–300 individuals (8). There is evidence for higher prevalence rates of HeFH in founder subpopulations like Saudi Arabians, in which consanguineous marriages are practiced as part of a social norm. Furthermore, FH is underdiagnosed all around the world, with <5% of affected individuals in many countries being identified as having FH (12). Owing to the limited data describing the genetic and phenotypic characteristics of hypercholesterolemia among Saudi patients (13–15) this study aims at identifying the inherited basis of FH in two consanguineous families from Saudi Arabia. In this study, we show that LipidSeq targeted resequencing panel for monogenic dyslipidemias, can effectively detect FH causative *LDLR* founder variant (c.2027delG) in genetically isolated populations like Saudi Arabians.

## Materials and Methods

### Recruitment of FH Patients and Their Families

The institutional Ethics Committee for Human Research of King Abdulaziz University Hospital (KAUH) gave the approval to conduct the present study according to standard international guidelines. This study has recruited FH patients from Genetic Dyslipidemia and Familial Hypercholesterolemia clinic at the King Abdulaziz University Hospital, Jeddah, Saudi Arabia. Initially two families with multiple members, fulfilling the combined FH diagnostic criteria of Simon Broome Register, and the Dutch Lipid Clinic Network (DLCN), were identified. In Simon Broome criteria for FH diagnosis, points are assigned for cholesterol concentrations, clinical characteristics, molecular diagnosis, and family history, which include risk of fatal heart disease (16). Although the Simon Broome Register criteria consider the molecular diagnosis as evidence for definite FH, the DLCN requires that at least one other criterion be met in addition to molecular diagnosis (17). All the affected individuals from these families underwent detailed physical examinations and their full family history was collected. Laboratory investigations for multiple parameters including Plasma lipid profile (LDL-C, HDL-C, Triglyceride and Total Cholesterol), blood glucose, thyroid function, and liver function were measured by a homogenous enzymatic assay. Clinical geneticist revisited the medical data of patients, interviewed them, drew three generation pedigree charts and enrolled the remaining relatives of the patient families. We have also recruited 92 unrelated FH patients following the DLCN criteria. Approximately 5 mL of blood sample (in EDTA vacutainers) was collected from all individuals after explanation of the study, along with risks and potential benefits. All the participants have signed the informed consent.

### Genotyping

#### DNA Preparation

Genomic DNA from peripheral blood cells was isolated using the standard protocols supplied by commercial extraction kits. DNA's quality and quantity were assessed with Nanodrop spectrophotometer and DNA integrity was checked with 1% agarose gel. DNA dilutions at starting concentration of 2 ng/μL were prepared with help of a Qubit 2.0 fluorometer.

#### Targeted DNA Resequencing With LipidSeq

The DNA samples of the index cases and other members from both families were sequenced on LipidSeq, a targeted resequencing panel for monogenic dyslipidemias, at London Regional Genomics Center, London, Ontario, Canada (www.lrgc.ca). This LipidSeq resequencing panel can scan pathogenic mutations in 73 genes and 185 single nucleotide polymorphisms (SNPs) associated with dyslipidemia and other metabolic disorders (18). A latest article has reviewed the utility of LipidSeq technology in successfully diagnosing the monogenic dyslipidemias and metabolic disorders (19). The full details of DNA library preparation, sequencing, sequence alignment and variant calling (10-fold coverage and 20% read frequency) are described in the original publication (18). VarSeq software was used to annotate and prioritize the variants and for identifying the FH potential variants. Different nucleotide sequence-based prediction algorithms, such as CADD, SIFT and PolyPhen-2 which assess pathogenicity of variants were used to filter the likely deleterious variants (20). The minor allele frequency (MAF) of the variants was determined based on the data available like SHGP, 1,000 genomes, ExAC, ESP and GnomAD databases. From this data, we picked up the extremely rare (MAF is <0.01) mutation occurring in coding regions or splicing regions of the FH causative disease genes and validated its presence in remaining family members and unrelated FH cases.

#### Sanger Sequencing

The LipidSeq identified a potential FH causative variant was validated in index case, family members and unrelated FH cases, using the Sanger sequencing method. In brief, initially oligonucleotide primers (forward primer; 5′-CCCAACCTTGAAACCTCCTTGTGGAAA-3′ and reverse primer; 5′-CCATTTGACAGATGAGCAGAGAG-3′) spanning the potential mutation location were designed, followed by PCR reactions with dNTP and ddNTP mixture, and bidirectional sequencing in an automatic DNA sequencing machine. The sequence reads were analyzed with help of Bioedit program and nucleotide numbering of the mutations was done considering A of ATG code of mRNA sequence as the first nucleotide. Variant segregation in the family was determined by careful analysis of variant status in each family member.

### Computational Functional Analysis of Pathogenic Mutations

#### Functional Analysis of Pathogenic Mutation on RNA Structure

Studying the impact of pathogenic variants on the RNA secondary structures gives hint about its possible functional consequences. Thus, we used a RNA fold (http://rna.tbi.univie.ac.at/cgi-bin/RNAWebSuite/RNAfold.cgi) prediction tool which estimates the back bone traces and minimum free energy (MFE) value differences on the optimal secondary structure of RNA molecule (21). This tool intakes the native and wildtype RNA sequences in FASTA format and uses Mccaskill's algorithm (22) for computing the probabilities of base pairing matrix, partition function, and structure of centroid molecules. The output of RNA folding is an interactive string representation RNA secondary structure and a mountain plot showing the folding of energy differences between native and mutant sequences. MFE differences between native and mutant RNA structures were compared to estimate the effect of pathogenic variant on their secondary structural features.

#### Functional Analysis of Pathogenic Mutation on Protein Structure

Studying the impact of pathogenic variants on protein structure provides insight into the complex dynamics of genotype vs. protein phenotype and structure-function relationships. In this study, we retrieved the x-ray crystallography solved tertiary structure of the query protein from Protein Data Bank (PDB). The construction of missing structural regions basing on original crystal structure coordinates was simulated through *ab-initio* method using I-Tasser webserver (23, 24). The full length tertiary model was subsequently processed for energy minimization and stereochemical assessment steps as described in our recent publication (25). We subsequently created mutant form of FH candidate protein by providing the mutant amino acid sequence and followed the similar steps involved in native protein structure modeling. The built 3D models were provided as an input to PDBSUM for examining the variant induced protein phenotype changes at secondary structure level. PyMOL software was used for visualizing and examining the salt bridges in, which the 3D models built. Stability changes induced by the variants on *LDLR* structure were estimated with help of DUET webserver (26).

## Results

### Case Presentations in the FH Families

#### Family A

**Figure 1:**
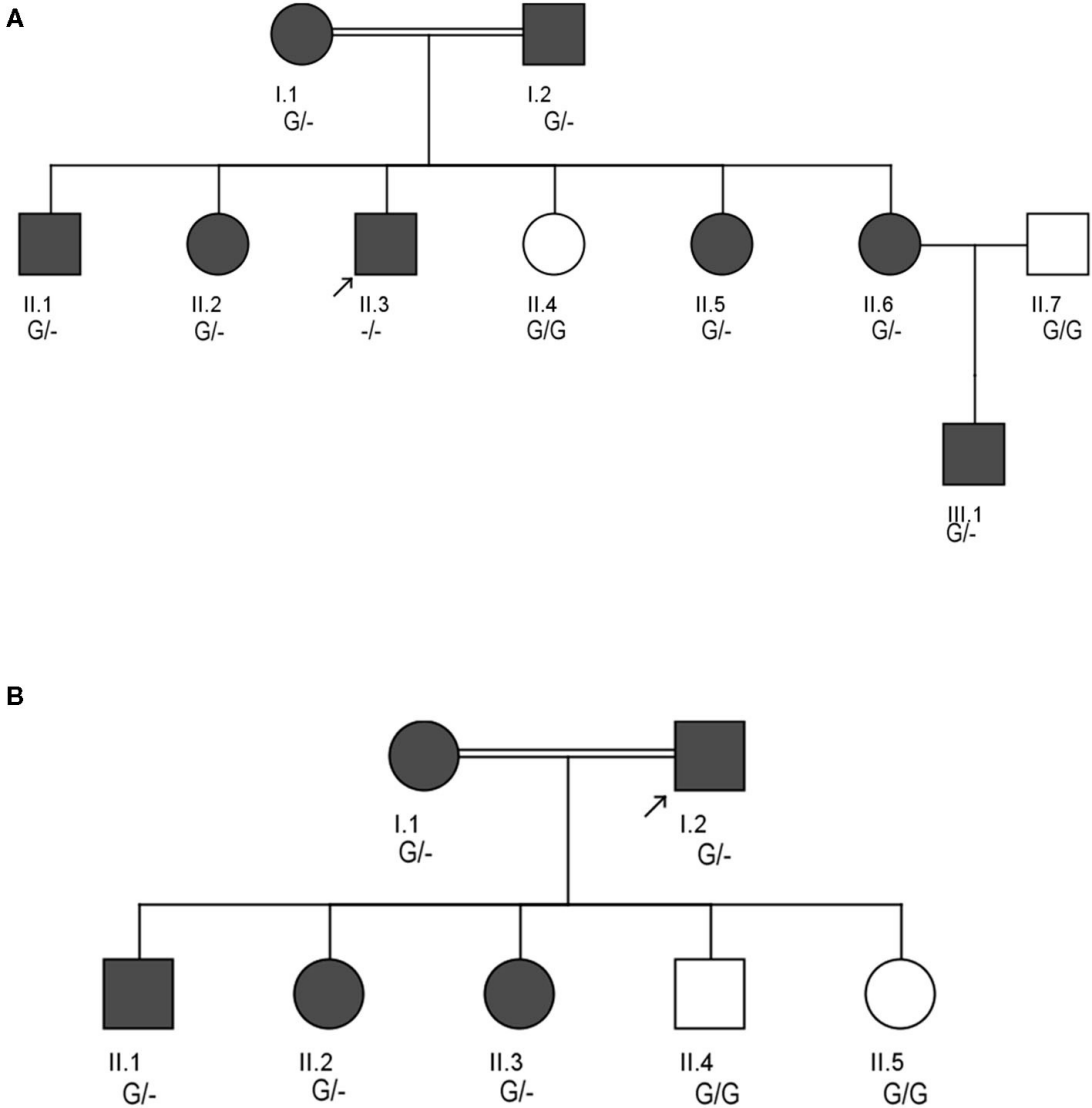
Pedigrees showing the autosomal dominant inheritance mode for *LDLR* variant (c.2027delG) in two different Saudi FH Families **(A,B)**. Arrow indicates the index case who was first seen in our clinic. The zygosity of the variant's genotype is mentioned under the subjects. Dark color circles or boxes in the pedigree indicates subjects with FH.

**Figure 2:**
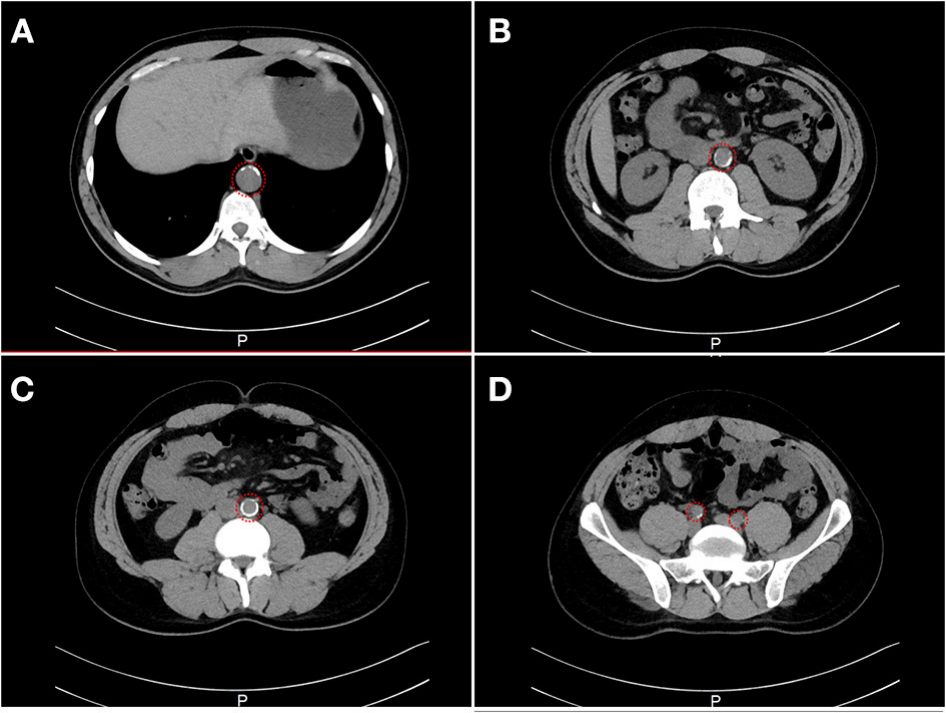
A computerized tomography (CT) scan showing the evidence of stenosis. CT cross-section of the thoraco-abdomen illustrating **(A)** Calcification within the aortic wall in the thoracic region (T11–T12). **(B)** Calcification within the aortic wall in the lumber region (L1–L2). **(C)** Calcification within the aortic wall in the lumber region (L4–L5). **(D)** Calcification within the iliac artery in the sacral region.

Family A is a native Saudi Arabian family from the North-Western region (Figure 1A). The index case (II.3) was clinically diagnosed as FH patient at the age of nine and later presented to our clinic when he was 34 years old for his lipid management. His clinical examination revealed signs of severe hyperlipidemia. All classical manifestations of FH were present including bilateral large Achilles tendon xanthomas, huge cholesterol depositions around both mid-thighs, severe bilateral eye xanthelasma and corneal arcus, and bilateral multiple extensor tendon xanthomas on hands. His biochemical profile revealed on an average high level of total cholesterol (15.18 ± 1.33 mmol/L), LDL-C (12.98 ± 2.08 mmol/L) and normal triglycerides (0.81 ± 0.16 mmol/L) (Table 1). He had a past history of hospitalization after chest discomfort and shortness of breath, during which electrocardiogram generated ischemic changes were noticed. A computerized tomography (CT) of the entire aorta was performed, which showed extensive atherosclerotic calcifications in the thoracic aorta, abdominal aorta and into the iliac vessels (Figure 2).

**Table 1:** Clinical and biochemical characteristics of FH families studied in this investigation.

**Family**	**Member**	**Genotype**	**LDL-C (mmol/L)**	**Reference rane**	**TC (mmol/L)**	**Reference range**	**TG (mmol/L)**	**Reference range**	**Clinical phenotype**
			**Mean ± Standard deviation**		**Mean ± standard deviation**		**Mean ± standard deviation**		
A	I.1	G/−	8.3 ± 1.98	**0–3.57 (mmol/L)**	11.0 ± 1.01	**0–5.20 (mmol/L)**	0.76 ± 0.29	**0.3–2.30 (mmol/L)**	Diabetes, hyperlipidemia, bilateral tendon xanthomas, history of atherosclerosis myocardial infarctions (MI).
	I.2	G/−	9.7 ± 1.32		13.7 ± 1.37		0.86 ± 0.41		Hyperlipidemia, bilateral tendon xanthomas, history of peripheral atherosclerosis in both legs and history of cardiovascular disease.
	II.1	G/−	11.4 ± 0.54		12.8 ± 0.91		0.66 ± 0.54		Hyperlipidemia, history of MI.
	II.2	G/−	10.1 ± 1.11		11.9 ± 1.11		0.78 ± 0.37		Hyperlipidemia, history of MI.
	**II.3**	−/−	13.4 ± 2.11		15.5 ± 1.24		0.82 ± 0.19		Statin resistance, bilateral xanthelasma, corneal arcus, bilateral tendon xanthomas, Achilles tendon xanthomas, severe and huge cholesterol depositions around both mid-thighs.
	II.4	G/G	–		–		–		–
	II.5	G/−	7.8 ± 0.98		14.6 ± 0.93		0.77 ± 0.33		Hyperlipidemia
	II.6	G/−	8.2 ± 1.22		12.8 ± 0.87		0.87 ± 0.23		Hyperlipidemia
B	I.1	G/−	7.6 ± 2.40		9.6 ± 1.33		0.75 ± 0.56		Hyperlipidemia, history of aortic atherosclerosis
	I.2^*^	G/−	2.2 ± 1.08		3.6 ± 1.24		1.69 ± 0.55		Sever aortic stenosis, multiple MI events and CABG surgery.
	II.1	G/−	4.7 ± 1.51		6.3 ± 1.81		1.73 ± 1.01		Hyperlipidemia, chronic angina and severe chest pain.
	II.2	G/−	3.8 ± 1.41		5.5 ± 1.46		0.86 ± 0.05		Diabetes, hyperlipidemia, chronic angina and severe chest pain.
	II.3	G/−	4.8 ± 2.26		6.3 ± 2.42		1.08 ± 0.09		Hyperlipidemia, chronic angina and severe chest pain.

* *On-treatment lipid measurements; G/G, homozygote, G/−, heterozygote, −/−, homozygote for LDLR, c.2027delG mutation*.

At the time of his visit to our lipid clinic, II.3 was on the following regimen; dual antiplatelet agents, angiotensin converting enzyme (ACE) inhibitors, β-blockers, ezetimibe 10 mg/day and intensive statin treatment with rosuvastatin 40 mg/day, without showing any signs of improvement in his lipid profile (and even when PCSK9 inhibitors were given). He has been undergoing a bimonthly LDL- apheresis therapy in the Cardiovascular Prevention and Rehabilitation Unit of a major referral hospital in Saudi Arabia over the last 10 years. The patient reported his strict adherence to diet and medications as per the physician's instructions. With each apheresis session, his LDL cholesterol level drops by 70 to 83 percent, but within 1 week, returns back to the pre-apheresis level.

Pedigree analysis of the index case suggested positive family history of dyslipidemia, consistent with an autosomal dominant mode of inheritance. The biochemical findings of his parents (I.1 and I.2), five siblings including two brothers (II.1 and II.3), and 3 sisters (II.2, II.6 and II.6) were consistent with an FH diagnosis. However, one sister (II.4) has showed a healthy lipid profile and normal clinical features. Clinical and medication details of family members are shown in Table 1. Furthermore, proband has reported that three of his grandparents (both maternal and paternal), two maternal uncles, three paternal uncles, and three siblings suffered from cardiovascular complications including multiple myocardial infarctions (MI). Of the three paternal uncles, two had percutaneous coronary interventions with insertion of coronary stents; one underwent coronary artery bypass grafting (CABG) in his forties.

#### Family B

The second consanguineous family comes from the Southern region of Saudi Arabia (Figure 1B). The proband (I.2) was referred to our clinic after undergoing CABG surgery together with replacement of two valves. His past medical history revealed that he was hypercholesterolemic since early adulthood, has undergone cardiac catheterization four times, and had multiple stent placements at the ages of 39 (1 stent), 42 (4 stents), and 44 (1 stent) due to coronary artery narrowing. At the age of 47, the patient was admitted for an open-heart surgery to perform CABG to improve blood flow and oxygen supply to the heart. Clinical examination did not reveal the presence of severe physical signs including the absence of Achilles and tendon xanthomas. The only physical finding was the presence of mild corneal arcus. At the time of his presentation at the lipid clinic, his on-treatment lipid measurements were as follows; total cholesterol 3.86 mmol/dL, LDL-C 2.69 mmol/Dl, and triglyceride level 1.13 mmol/L (Table 1). Despite receiving combination of lipid lowering drugs i.e., ezetimibe 10 mg daily, and evolocumab subcutaneous injections 140 mg/mL once every 2 weeks, there was no improvement in his blood LDL cholesterol, which ranged between (2.47–3.09 mmol/L).

The clinical screening, biochemical investigations and pedigree analysis of this family were consistent with an autosomal dominant mode of inheritance. As per biochemistry reports, spouse of the index case (I.1), elder son (II.1) and two daughters (II.2 and II.3) were also dyslipidemic. However, his younger son (II.4) and younger daughter (II.5) were healthy and free from any symptoms related to dyslipidemia (Table 1). The index case (I.2) and his wife (I.1) both have reported that their mothers have died before the age of 60 due to myocardial infarction (MI) and other heart associated related complications. Moreover, the elder sister of the index case (I.1) and younger brother of the spouse (I.2) were reported to have had open-heart surgery before their fifties due to severe MI after they had cardiac catheterizations initially at the age of 25. All these cardiac events in the family and elevated blood lipid profiles strongly suggests premature atherosclerosis which is consistent with severe heterozygous or homozygous FH.

### Genetic Analysis

**Figure 3:**
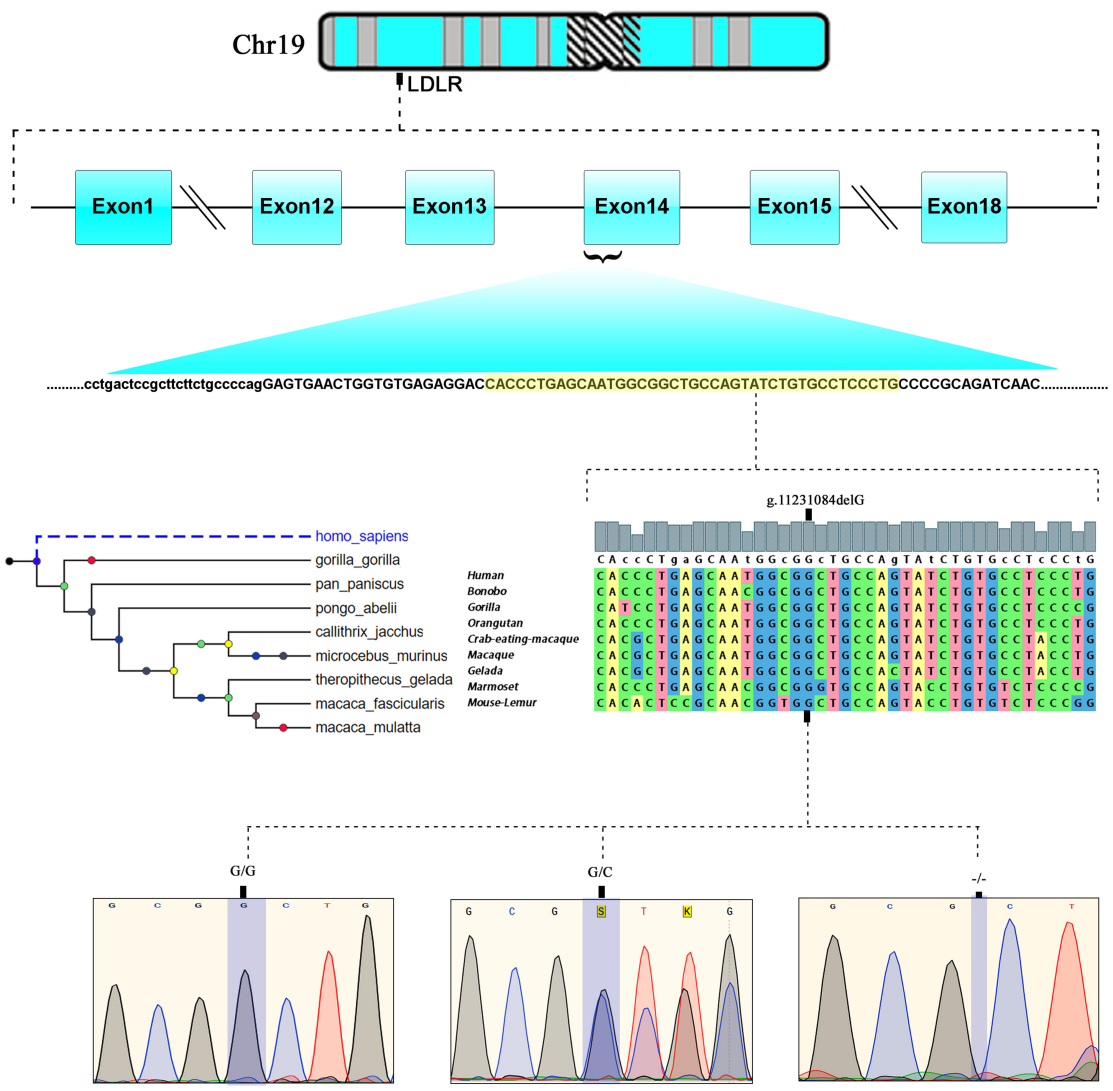
Chromosomal location of human *LDLR* gene at chromosome19p13.2. exonic position and multiple sequence alignment showing the *LDLR*, EGF like domain sequence across different mammalian species and chromatograms of c.2027delG variant showing wild type (GG), heterozygote (G/−), and homozygous mutant (−/−) genotypes.

The LipidSeq data of both families were analyzed for pathogenic mutations in *LDLR, APOB, PCSK9, ARH, APOE, ABCG5, ABCG8, and LIPA* owing to their known involvement in FH. Out of all FH candidate genes screened, only one a rare pathogenic c. 2027delG (g.11231084delG) variant localized to exon 14 of the *LDLR* gene, which is positioned on chromosome 19 p13.2 was noticed in 8 affected individuals in family A and five individuals in family B. This deletion mutation results in a frameshift in coding sequence of the *LDLR* gene and subsequently substitutes the native amino acid glycine to variant alanine at 676th position, followed by 33 nonsense residues, leading eventually to a premature stop gain signal to truncate the *LDLR* protein (UniProtKB - P01130) at 709th amino acid (G676AfsX33). This variant is expected to result in a prematurely truncated protein which likely undergoes nonsense-mediated protein decay (Figure 3).

This *LDLR* variant is listed in both dbSNP (ID: rs875989937) and ClinVar (ID: 226383). The rare prevalence of this variant is also ascertained through its absence in Exome Aggregation Consortium (ExAC), 1,000 genomes, EURO-WABB (LOVD), Greater Middle East (GME) Variome and Saudi Human Genome Program (SHGP) databases. This variant has a very low frequency of 0.0001 (six homozygotes and one heterozygote out of 4,706 individuals) in Saudi population as per the Saudi Human Genome Program database. According to variant interpretation standards and guidelines set out by American College of Medical Genetics and Genomics (ACMG), this variant is very strongly predicted to be pathogenic because it's a null variant in coding region of the *LDLR* gene whose loss of function (LOF) is a well-known mechanism for FH. Moreover, this mutation has been reported to fully segregate with FH in few Saudi families (27, 28) and is also listed in Human Genome Mutation Database (HGMD) to cause FH. Sanger sequencing results have confirmed the autosomal dominant mode of inheritance of variant in FH patients from both families. The distribution of mutation in family A is as follows; both parents (I.1 and I.2), four siblings (one brother- II.1 and three sisters- II.2, II.5 and II.6) were heterozygote carriers of the variant. Whereas, the index case (II.3) was homozygote for the c. 2027delG variant. As described in previous section, this index case has presented with severe clinical features of FH, including Achilles tendon xanthomas. However, the younger sister (II.4) of index case was homozygous for the reference G allele. In family B, the proband (I.2), his wife (I.1), elder son (II.1) and two daughters (II.2 and II.3) were heterozygotes, whereas his younger son (II.4) and younger daughter (II.5) were homozygote carriers of the reference G allele. The genotyping results in both families corroborate with the biochemical and clinical findings. This frameshift deletion variant was found to be completely absent in an unrelated 92 FH cases tested in this study, which suggests a strong possibility that c.2027delG of the *LDLR* gene is a potential FH founder mutation in Saudi patients. Furthermore, this variant was seen to be located in evolutionarily highly conserved region of gene sequence across different species like *Gorilla, Panicus, Pongo abelli, Callithrix jachchus, Microcebus murinus, Theropithecus gelada, Macaca fascicularis, Macaca mulatta* etc.

### Computational Functional Analysis of Pathogenic Mutation

#### Functional Impact of Pathogenic Mutation on RNA Structure

**Figure 4:**
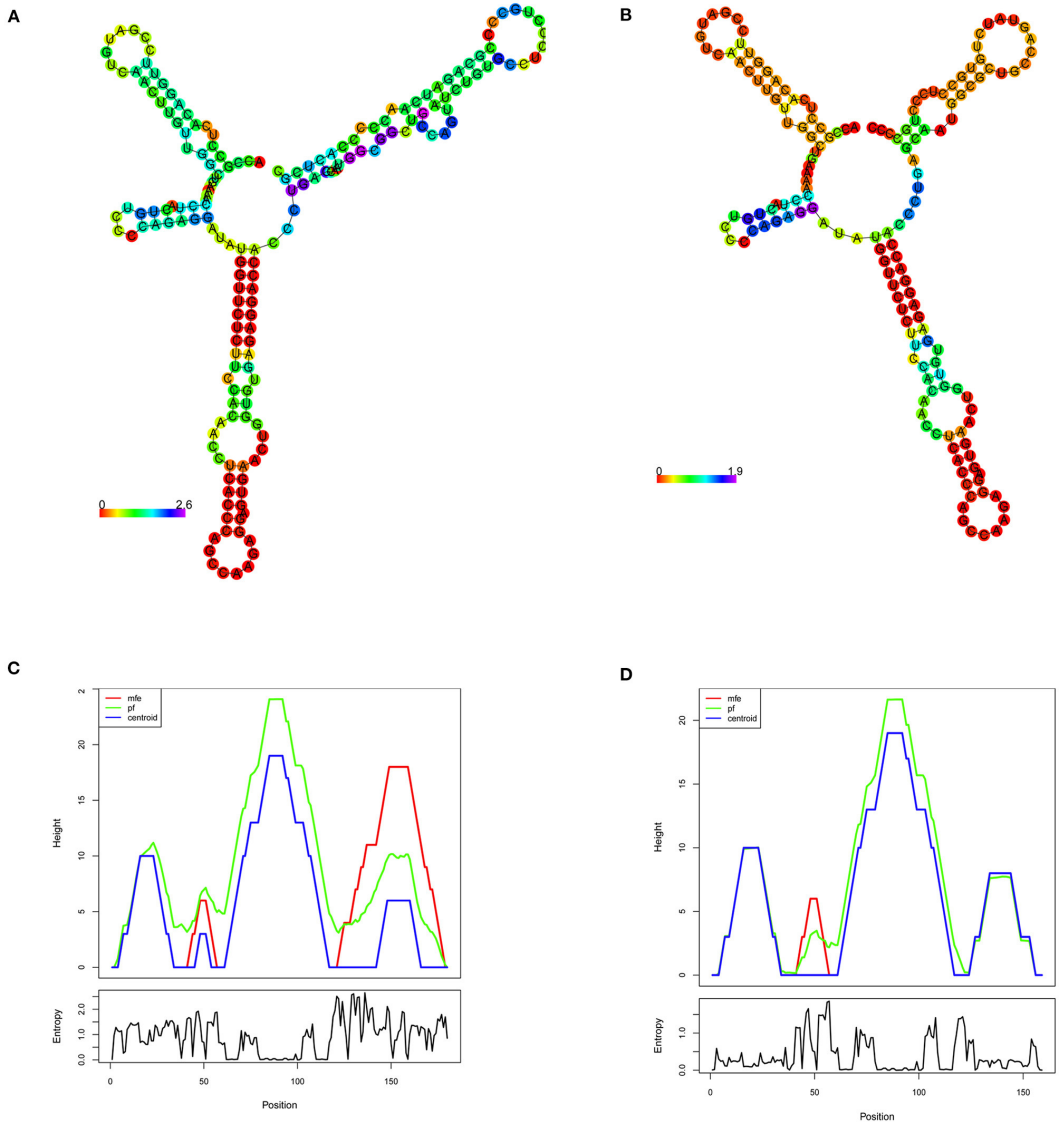
RNA secondary structure prediction of the human *LDLR* by RNA Fold. **(A,B)** Shows the *LDLR*, RNA secondary structure predictions for wildtype and mutant (c.2207delG), respectively, based on minimum free energy (MFE) calculations of nucleotide base pairing, which is represented by color gradient in the scale of 0–2. **(C,D)** Shows the mountain plot (MP) representation of MFE, thermodynamic ensemble (pf) and the centroid structure predictions, of the *LDLR* native and mutant (c.2207delG) RNA secondary structures, respectively. MP shows the secondary structures in a height vs. position, where the helices are represented in slopes, loops in plateaus and hairpin loops in the peaks. The bottom graph represents the entropy of predicted RNA structure, where higher the entropy means the RNA structure has lower stability.

The minimum free energy (MFE) calculation of *LDLR* centroid structures revealed that mutant mRNA molecule of *LDLR* (c.2027delG) possesses a relatively lower stability of secondary structures with −50.70 kcal/mol compared to native *LDLR* mRNA molecules (MEF was −52.80 kcal/mol). Hence, it is assumed that the lower stability of mRNA with c.2027delG is likely to affect the mRNA folding pattern and tertiary structure formation (Figure 4).

#### 3-Dimensional (3D) Protein Modeling and Secondary Structure Analysis

**Figure 5:**
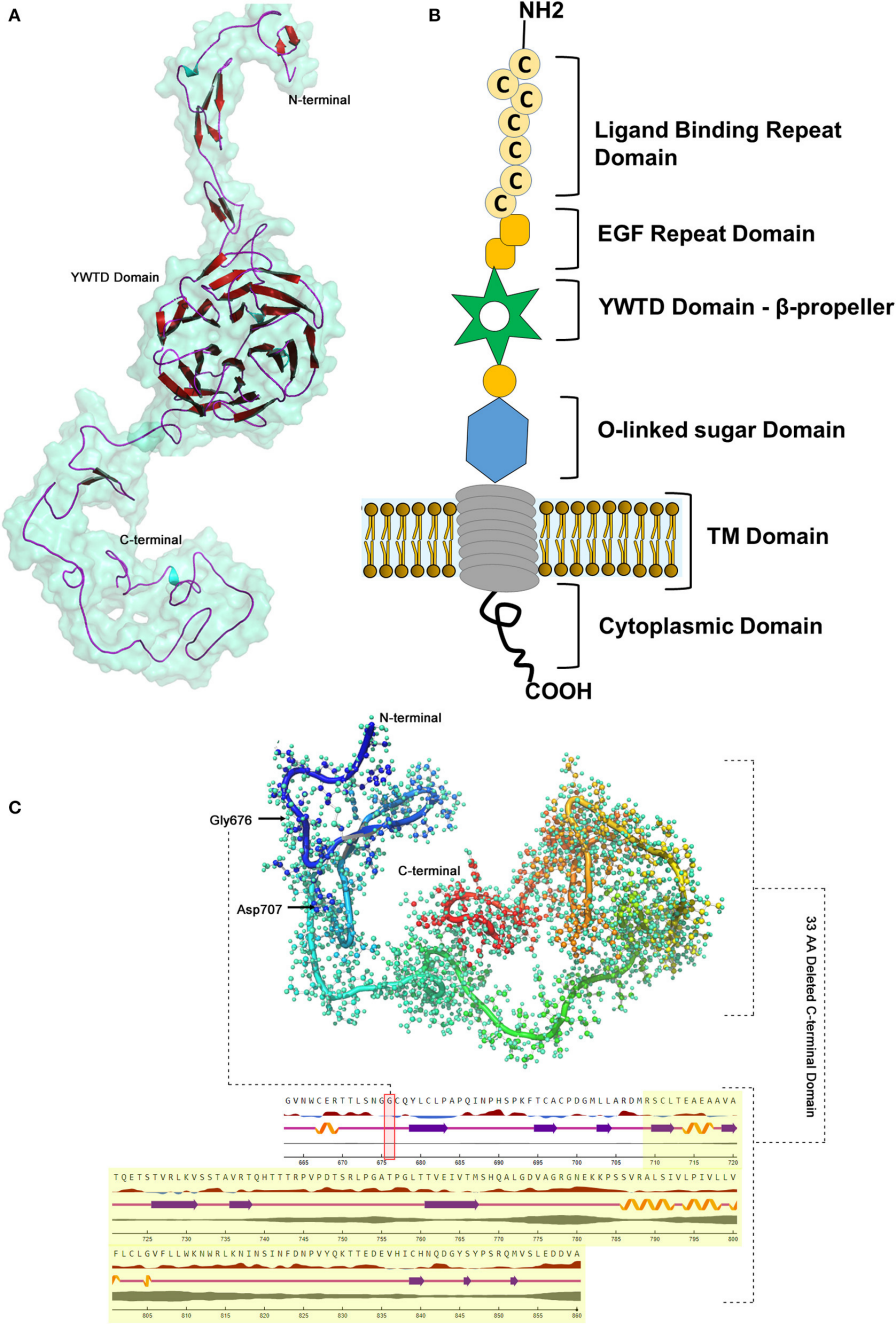
*LDLR* protein structure visualization. **(A)** 3D structural representation of the protein molecule. **(B)** Functional domains distribution. **(C)** The p.G676Afs*33 variant effect on the secondary structure organization on the protein.

The BLASTP program search for *LDLR* protein (860 aa) identified that the PDB sourced experimentally solved structure (3M0C: chain C) has 91% (1–715 aa) of amino acid sequence coverage. However, the remaining 9% of the sequence spanning 786–860th amino acids is not yet solved. The structure of *LDLR* spans over LD repeat domain (20–311), EGF like domain (314–712), oligosaccharide linked sugars (700–758), membrane domain (residues 759–781) and cytoplasmic domain (811–860). Hence, the missing chain portions from EGF like and cytoplasmic domains were simulated by using I-Tasser, which predicted 5 probable models. The best fit *LDLR* model was selected based on confidence (1.25), template modeling (0.54 ± 0.12) and root mean square deviation scores (4.5 ± 2.8). The built protein models were subsequently energy minimized and taken as reference in constructing mutant model, which were later used to predict the effect of variant on secondary structural features. The native *LDLR* secondary structure is characterized by 3 α-helices, 11 sheets, 164 β-strands (11 β-sheets, 22 β-hairpins, 20 β-bulges, 77 β-turns, 34 β-pleated strands), 12 loops (12 γ-turns) and 243 other components like disulphide bridges. The G676A missense variant is localized to 3rd helix, does not change the secondary structure conformation as such, but truncation of the protein at Asp 707 residue eliminates/skips 34th β-strand and 12th loop spanning from 714 to 860th amino acid toward C-terminal region of the protein (Figure 5).

## Discussion

FH occurs in two clinical forms, namely homozygous or heterozygous, depending upon the gene dosage of the variant alleles, i.e., bi-allelic and mono-allelic, respectively (29). In the current study, we identified both homozygous (one patient) and several heterozygous FH patients (12 patients) from two unrelated Saudi Bedouin families bearing a pathogenic c.2027delG (p.G676Afs^*^33) stop gain mutation. This mutation was reported as c.2026delG in 5 among 4 HoFH patients and 14 HeFH patients belonging to different tribes from Saudi Arabia (27, 28). So far, five different *LDLR* mutations (p.D445^*^, p. R471R, p. G676Afs^*^33, p.Y419D, p.W577^*^) were identified in 27 FH patients from five studies from Saudi Arabia (Table 2). Hence, most likely c.2027delG is the founder FH mutation in Saudi population, where both inter- and intra- consanguineous marriages among tribal communities is a normal practice. A few other FH founder mutations in the *LDLR* gene have been previously identified among French Canadians from Quebec Province (33–35), Finnish from Finland (36) and Dutch from Netherlands (37).

**Table 2:** *LDLR* variants reported in Saudi FH patients.

**S.No**	**Variant ID**	**Nucleotide change**	**Amino acid change**	**Exon**	**Variant Nature**	**No of Patients**	**FH Zygosity**	**Reference**
1	–	c.1332dupA	p.D445*	9	Missense	2	HoFH	(30)
	rs5930	c.1413A>G	p.R471R	10	Silent	2	HoFH	
2	–	c.2026delG	p.G676Afs*33	14	Frameshift	1	HeFH	(27)
3	–	c.2027delG	p.G676Afs*33	14	Frameshift	11	HeFH	(28)
					Frameshift	9	HoFH	
	rs5930	c.1413A>G	p.R471R	10	Silent	8	HeFH	
4	–	c.1255T>G	p.Y419D	9	Missense	1	HoFH	(31)
5	–	c.1731G>T	p.W577*	12	Missense	3	HeFH	(32)
6	–	c.2027delG	p.G676Afs*33	14	Frameshift	11	HeFH	Present Study
					Frameshift	1	HoFH	

In the current study, we noticed that a clinically severe patient with a phenotype resembling HoFH (II.3) from family A, has inherited two copies *LDLR* c.2027delG stop gain mutation from his HeFH parents (i.e., bi-allelic), although one defective copy is sufficient enough to develop the disease in heterozygotes. The severe clinical signs observed in this patient could undoubtedly be the result of an extremely compromised receptor capacity, a null variant with <2% functional activity, therefore homozygosity would explain their particularly severe biochemical and clinical phenotype. The LDL-C levels in HoFH due to bi-allelic null variants can increase by 4 to 10-fold from normal (38). It is notable that this HoFH patient despite having severe coronary stenoses, has not yet reported any cardiovascular events like myocardial infarctions (MI). Although this HoFH patient has presented with severe atherosclerosis, we speculate that early disease diagnosis (9 years), continuous medications, regular clinical monitoring and lifestyle modifications, would have averted the possibility of severe or fatal cardiac event. Literature review suggests that untreated HoFH patients by their 2nd decade of life present with CVD due to the development of advance atherosclerotic plaques and stenosis in blood vessels (38). A clinical assessment and follow-up study of 39 HoFH patients under <16 years of age reported cardiovascular events in 88% of the subjects (39). The HoFH patients show worse prognosis even on maximum treatment doses of lipid lowering drugs these patients show LDL-C levels >7.8 mmol/L. Our HoFH patient has also manifested tendon xanthomas which are known to be formed by huge cholesterol depositions in the tendons and joints that may account for pain and disability (38). Arcus cornealis, are bright zone of cholesterol deposits around the rim of the cornea before the age of 40 and is an additional clinical feature observed in HoFH patients (40).

Most FH patients from both family (A and B) are heterozygous and carry one copy of the *LDLR*, c.2027delG stop mutation, which could have been inherited from either of their parents following an autosomal dominant mode of inheritance. These patients demonstrate 2/3rd reduction in LDL clearance rate, which subsequently elevates the circulating LDL-C by 2 to 3-folds (5–10 mmol/L; 200–400 mg/L) (10, 29, 41). FH patients manifest the disease in their adulthood, spanning 3^−^7th decades of their life (10, 41). We have observed that the majority of our HeFH patients have presented with MI before their 60th birthday. These clinical signs point us toward chronic deposits of cholesterol that induce arterial atherosclerotic damages. Careful physical examination in childhood often could prompts the early clinical diagnosis of HoFH. The undiagnosed and untreated HeFH patients have a very high risk of (10 to 20-folds) of developing premature coronary artery disease (CAD) (42), while the risk in untreated HoFH can be 100 to 200-fold increased from normal (38). The variable expressivity of FH can be attributed to modifier variants in LDLRAP1, *EPHX2, ABCG5, ABCG8, LIPA*, or *APOE* genes or polygenic risk variants (2, 43, 44). Early intervention to control the high LDL-C levels is clearly beneficial in reducing the cardiovascular events among young FH patients (45). The genetic testing of *LDLR*, c.2027delG variant in extended family members of both family A and B could potentially offer an advantage of early identification of FH cases, planning lifelong lipid lowering therapy, genetic counseling, and prenatal diagnosis (46).

The *LDLR* allelic makeup determines the molecular diagnosis (i.e., heterozygous vs. homozygous) and in turn determines the severity of clinical manifestation in FH. HoFH patients who have lost most or all receptor function, show very high circulating LDL-C levels and manifest cholesterol deposits in the body compared to HeFH patients who can still maintain 50% of functional receptors (47–49). Deleterious *LDLR* mutations are known to either eliminate or considerably reduce the LDLR function (50). This is true for the c.2027delG (p.G676Afs^*^33) frame shift mutation identified in this study, that introduces a protein termination codon (PTC) at 33rd codon downstream, and leads to the truncation of the LDLR protein at the Asp709th residue located in *EGF like domain*. The truncated protein will be 152 amino acid (aa) shorter than the native LDLR and lacks 5 aa residues from the *EGF like domain*, 58 aa residues of oligosaccharide linked sugars domain and 22 aa residues of the receptor transmembrane domain. Spanning between 314 and 712 aa is the *EGF like domain* the largest LDLR protein domain where more than 50% of FH causative mutations are reported highlighting its functional importance. EGF homology domain controls the release of lipoproteins in low pH environment and take part in receptor recycling (51). It is therefore fair to assume that the truncated *LDLR* may undergo degradation that may reduce the LDLR protein level and subsequently interfere with the receptor assembly in those individuals who harbor the mutation.

Indeed, the mRNAs with PTC may potentially activates nonsense mediated mRNA decay (NMD) (52) or leave a markedly truncated LDLR protein (at variant residue 709) which may eventually undergo degradation by ubiquitin mediated proteasomal pathway (53). The correlation between *LDLR* variant zygosity and its effect on protein expression is shown through functional biology experiments on lymphocytes obtained from FH patients with nonsense mutations (W23X, S78X, E207X, and W541X) (52). Human mRNAs with PTC were reported to reduce both mRNA abundance and stability (54). Our free energy-based RNA stability investigation predicted that the *LDLR*, p.G676Afs^*^33 variant destabilizes the mRNA structure, and eventually affect its folding pattern. Free energy changes are also affect the protein structure stability for disease causative stop gain mutations (55). Therefore, we predict that p.G676Afs^*^33 is a loss-of-function (LoF) variant or null allele leading to abolition of LDLR protein synthesis, hence it belongs to *LDLR* class 1 category of mutations (56).

Recent advances in molecular and cell biology present an alternative therapeutic opportunity to counter the genetic defects (PTC mutations) by pharmacological modulation (NMD or proteasomal degradation inhibitors) and help to control disease pathogenesis (54, 57, 58). In theory, HoFH patients who are unresponsive to statins might benefit from the use of pharmacological modulators like aminoglycosides in restoring partial sized LDLR molecules arising from stop gain mutations. In experiments, treating lymphocytes bearing different nonsense FH mutations with different translation modulator drugs, Holla *et al* has successfully demonstrated the increased mRNA levels of *LDLR* and LDL-C clearance (52). There are numerous other treatments in development for severe HeFH and HoFH, including inhibitors of angiopoietin like 3 protein (ANGPTL3), long-acting inhibitors of proprotein convertase subtilisin/kexin type 9 (PCSK9) in addition to gene therapies (59).

In conclusion, this study reports a very rare, pathogenic and FH founder *LDLR* stop gain variant i.e. c.2027delG (p.Gly676Alafs^*^33) in 12 FH patients belonging to two different Saudi families. Founder FH mutations concentrate due to low genetic variability in genetically isolated populations. Nevertheless, identifying FH founder confer advantages like targeted screening, early genetic diagnosis, genetic counseling and adoption of effective anti-lipid treatment strategies (i.e., LDL-apheresis) to prevent the cardiovascular disease burden among the FH patients. Based on the variant zygosity of the stop gain variant, we have noticed marked phenotypic heterogeneity in terms of LDL-C levels and clinical presentations of the FH patients. This loss-of-function variant was predicted to alter the free energy dynamics of RNA molecule hence its stability. Protein structure mapping has predicted that this variant eliminates key *LDLR* functional domains and eventually undergoes degradation. However, future functional biology studies are required to study the effect of c.2027delG variant on LDL-C clearance in the body.

## Data Availability Statement

The datasets presented in this article are not readily available because (a) participants' refusal to store or distribute the genomic data in the public domain and (b) as per the local Institutional Ethics committee approval and Saudi national policy on genomic data sharing in the public domain outside the country. Requests to access the datasets should be directed to NS or BB.

## Ethics Statement

The studies involving human participants were reviewed and approved by Ethics Committee for Human Research of King Abdulaziz University Hospital. Written informed consent to participate in this study was provided by the participant and legal guardian/next of kin of the children below 16 years.

## Author Contributions

ZA, BB, RE, JA-A, and NS: conceptualization. OR, NS, and BB: data curation. RH, ZA, and OR: formal analysis. NS: funding acquisition and project administration. OR, RH, BA-S, NS, and BB: methodology. BB: software. ZA, RH, NS, BB, and RE: supervision. OR and KJ: validation. BB: visualization. ZA, OR, RE, BB, NS, and RH: writing original draft and editing. All authors contributed to the article and approved the submitted version.

## Conflict of Interest

The authors declare that the research was conducted in the absence of any commercial or financial relationships that could be construed as a potential conflict of interest.

## References

[1] Bouhairie VE Goldberg AC. Familial hypercholesterolemia. Cardiol Clin. (2015) 33:169–79. 10.1016/j.ccl.2015.01.001 25939291 PMC4472364

[2] Trinder M Paquette M Cermakova L Ban MR Hegele RA Baass A. Polygenic contribution to low-density lipoprotein cholesterol levels and cardiovascular risk in monogenic familial hypercholesterolemia. Circ Genom Precis Med. (2020) 13:515–23. 10.1161/CIRCGEN.120.002919 33079599 PMC7889287

[3] Taylor B Cheema A Soslowsky L. Tendon pathology in hypercholesterolemia and familial hypercholesterolemia. Curr Rheumatol Rep. (2017) 19:76. 10.1007/s11926-017-0704-2 29101577

[4] Singh S Bittner V. Familial hypercholesterolemia–epidemiology, diagnosis, and screening. Curr Atheroscler Rep. (2015) 17:482. 10.1007/s11883-014-0482-5 25612857

[5] Bamimore MA Zaid A Banerjee Y Al-Sarraf A Abifadel M Seidah NG. Familial hypercholesterolemia mutations in the Middle Eastern and North African region: a need for a national registry. J Clin Lipidol. (2015) 9:187–94. 10.1016/j.jacl.2014.11.008 25911074

[6] Alallaf F Fa HN Alnefaie M Almaymuni A Rashidi OM Alhabib K. The spectrum of Familial Hypercholesterolemia (FH) in Saudi Arabia: prime time for patient FH registry. Open Cardiovasc Med J. (2017) 11:66–75. 10.2174/1874192401711010066 28868092 PMC5564019

[7] Turgeon RD Barry AR Pearson GJ. Familial hypercholesterolemia: review of diagnosis, screening, and treatment. Can Fam Physician. (2016) 62:32–7. 26796832 PMC4721838

[8] Berberich AJ Hegele RA. The complex molecular genetics of familial hypercholesterolaemia. Nat Rev Cardiol. (2019) 16:9–20. 10.1038/s41569-018-0052-6 29973710

[9] Paththinige CS Sirisena ND Dissanayake V. Genetic determinants of inherited susceptibility to hypercholesterolemia - a comprehensive literature review. Lipids Health Dis. (2017) 16:103. 10.1186/s12944-017-0488-4 28577571 PMC5457620

[10] Parihar RK Razaq M Saini G. Homozygous familial hypercholesterolemia. Indian J Endocrinol Metab. (2012) 16:643–5. 10.4103/2230-8210.98032 22837934 PMC3401774

[11] Mcgowan MP Hosseini Dehkordi SH Moriarty PM Duell PB. Diagnosis and treatment of heterozygous familial hypercholesterolemia. J Am Heart Assoc. (2019) 8:e013225. 10.1161/JAHA.119.013225 31838973 PMC6951065

[12] Nordestgaard BG Benn M. Genetic testing for familial hypercholesterolaemia is essential in individuals with high LDL cholesterol: who does it in the world? Eur Heart J. (2017) 38:1580–3. 10.1093/eurheartj/ehx136 28419271

[13] Mahzari M Zarif H. Homozygous familial hypercholesterolemia (HoFH) in Saudi Arabia and two cases of lomitapide use in a real-world setting. Adv Ther. (2021) 38:2159–69. 10.1007/s12325-021-01720-y 33829367 PMC8107066

[14] Albakheet N Al-Shawi Y Bafaqeeh M Fatani H Orz Y Shami I. Familial hypercholesterolemia with bilateral cholesterol granuloma: a case series. Int J Surg Case Rep. (2019) 62:135–9. 10.1016/j.ijscr.2019.07.018 31499414 PMC6734150

[15] Awan ZA Bondagji NS Bamimore MA. Recently reported familial hypercholesterolemia-related mutations from cases in the Middle East and North Africa region. Curr Opin Lipidol. (2019) 30:88–93. 10.1097/MOL.0000000000000586 30694837

[16] Henderson R O'kane M Mcgilligan V Watterson S. The genetics and screening of familial hypercholesterolaemia. J Biomed Sci. (2016) 23:39. 10.1186/s12929-016-0256-1 27084339 PMC4833930

[17] Austin MA Hutter CM Zimmern RL Humphries SE. Genetic causes of monogenic heterozygous familial hypercholesterolemia: a HuGE prevalence review. Am J Epidemiol. (2004) 160:407–20. 10.1093/aje/kwh236 15321837

[18] Johansen CT Dube JB Loyzer MN Macdonald A Carter DE Mcintyre AD. LipidSeq: a next-generation clinical resequencing panel for monogenic dyslipidemias. J Lipid Res. (2014) 55:765–72. 10.1194/jlr.D045963 24503134 PMC3966710

[19] Dron JS Wang J Mcintyre AD Iacocca MA Robinson JF Ban MR. Six years' experience with LipidSeq: clinical and research learnings from a hybrid, targeted sequencing panel for dyslipidemias. BMC Med Genomics. (2020) 13:23. 10.1186/s12920-020-0669-2 32041611 PMC7011550

[20] Wang J Dron JS Ban MR Robinson JF Mcintyre AD Alazzam M. Polygenic versus monogenic causes of hypercholesterolemia ascertained clinically. Arterioscler Thromb Vasc Biol. (2016) 36:2439–45. 10.1161/ATVBAHA.116.308027 27765764

[21] Lorenz R Bernhart SH Honer Zu Siederdissen C Tafer H Flamm C Stadler PF. ViennaRNA Package 2.0. Algorithms Mol Biol. (2011) 6:26. 10.1186/1748-7188-6-26 22115189 PMC3319429

[22] Mccaskill JS. The equilibrium partition function and base pair binding probabilities for RNA secondary structure. Biopolymers. (1990) 29:1105–19. 10.1002/bip.360290621 1695107

[23] George Priya Doss C Nagasundaram N Chakraborty C Chen L Zhu H. Extrapolating the effect of deleterious nsSNPs in the binding adaptability of flavopiridol with CDK7 protein: a molecular dynamics approach. Hum Genomics. (2013) 7:10. 10.1186/1479-7364-7-10 23561625 PMC3726351

[24] Thirumal Kumar D Jerushah Emerald L George Priya Doss C Sneha P Siva R Charles Emmanuel Jebaraj W. Computational approach to unravel the impact of missense mutations of proteins (D2HGDH and IDH2) causing D-2-hydroxyglutaric aciduria 2. Metab Brain Dis. (2018) 33:1699–710. 10.1007/s11011-018-0278-3 29987523

[25] Ahmed Awan Z Bima A Rashidi OM Jamil K Khan IA Almukadi HS. Low resolution protein mapping and KB-R7943 drug-protein molecular interaction analysis of long-QT syndrome linked KCNH2 mutations. All Life. (2020) 13:183–93. 10.1080/26895293.2020.1737249

[26] Shaik NA Bokhari HA Masoodi TA Shetty PJ Ajabnoor GMA Elango R. Molecular modelling and dynamics of CA2 missense mutations causative to carbonic anhydrase 2 deficiency syndrome. J Biomol Struct Dynam. (2020) 38:4067–80. 10.1080/07391102.2019.1671899 31542996

[27] Al-Allaf FA Athar M Abduljaleel Z Taher MM Khan W Ba-Hammam FA. Next generation sequencing to identify novel genetic variants causative of autosomal dominant familial hypercholesterolemia associated with increased risk of coronary heart disease. Gene. (2015) 565:76–84. 10.1016/j.gene.2015.03.064 25839937

[28] Al-Allaf FA Alashwal A Abduljaleel Z Taher MM Siddiqui SS Bouazzaoui A. Identification of a recurrent frameshift mutation at the LDLR exon 14 (c.2027delG, p.(G676Afs^*^33)) causing familial hypercholesterolemia in Saudi Arab homozygous children. Genomics. (2016) 107:24–32. 10.1016/j.ygeno.2015.12.001 26688439

[29] Hovingh GK Davidson MH Kastelein JJ O'connor AM. Diagnosis and treatment of familial hypercholesterolaemia. Eur Heart J. (2013) 34:962–71. 10.1093/eurheartj/eht015 23416791

[30] Al-Allaf FA Athar M Abduljaleel Z Bouazzaoui A Taher MM Own R. Identification of a novel nonsense variant c.1332dup, p.(D445^*^) in the LDLR gene that causes familial hypercholesterolemia. Hum Genome Var. (2014) 1:14021. 10.1038/hgv.2014.21 27081511 PMC4785512

[31] Alnouri F Athar M Al-Allaf FA Abduljaleel Z Taher MM Bouazzaoui A. Novel combined variants of LDLR and LDLRAP1 genes causing severe familial hypercholesterolemia. Atherosclerosis. (2018) 277:425–33. 10.1016/j.atherosclerosis.2018.06.878 30270081

[32] Al-Allaf FA Alashwal A Abduljaleel Z Taher MM Bouazzaoui A Abalkhail H. Compound heterozygous LDLR variant in severely affected familial hypercholesterolemia patient. Acta Biochim Pol. (2017) 64:75–9. 10.18388/abp.2016_1283 27878139

[33] Simard LR Viel J Lambert M Paradis G Levy E Delvin EE. The Delta>15 Kb deletion French Canadian founder mutation in familial hypercholesterolemia: rapid polymerase chain reaction-based diagnostic assay and prevalence in Quebec. Clin Genet. (2004) 65:202–8. 10.1111/j.0009-9163.2004.00223.x 14756670

[34] Leitersdorf E Tobin EJ Davignon J Hobbs HH. Common low-density lipoprotein receptor mutations in the French Canadian population. J Clin Invest. (1990) 85:1014–23. 10.1172/JCI114531 2318961 PMC296530

[35] Bétard C Kessling AM Roy M Chamberland A Lussier-Cacan S Davignon J. Molecular genetic evidence for a founder effect in familial hypercholesterolemia among French Canadians. Hum Genet. (1992) 88:529–36. 10.1007/BF00219339 1348044

[36] Lahtinen AM Havulinna AS Jula A Salomaa V Kontula K. Prevalence and clinical correlates of familial hypercholesterolemia founder mutations in the general population. Atherosclerosis. (2015) 238:64–9. 10.1016/j.atherosclerosis.2014.11.015 25437892

[37] Kusters DM Huijgen R Defesche JC Vissers MN Kindt I Hutten BA. Founder mutations in the Netherlands: geographical distribution of the most prevalent mutations in the low-density lipoprotein receptor and apolipoprotein B genes. Netherlands Heart J. (2011) 19:175–82. 10.1007/s12471-011-0076-6 21475731 PMC3058324

[38] Cuchel M Bruckert E Ginsberg HN Raal FJ Santos RD Hegele RA. Homozygous familial hypercholesterolaemia: new insights and guidance for clinicians to improve detection and clinical management. A position paper from the Consensus Panel on Familial Hypercholesterolaemia of the European Atherosclerosis Society. Eur Heart J. (2014) 35:2146–57. 10.1093/eurheartj/ehu274 25053660 PMC4139706

[39] Kolansky DM Cuchel M Clark BJ Paridon S Mccrindle BW Wiegers SE. Longitudinal evaluation and assessment of cardiovascular disease in patients with homozygous familial hypercholesterolemia. Am J Cardiol. (2008) 102:1438–43. 10.1016/j.amjcard.2008.07.035 19026292

[40] Kim YR Han KH. Familial hypercholesterolemia and the atherosclerotic disease. Korean Circ J. (2013) 43:363–7. 10.4070/kcj.2013.43.6.363 23882283 PMC3717417

[41] Mytilinaiou M Kyrou I Khan M Grammatopoulos DK Randeva HS. Familial hypercholesterolemia: new horizons for diagnosis and effective management. Front Pharmacol. (2018) 9:707. 10.3389/fphar.2018.00707 30050433 PMC6052892

[42] Goldberg AC Hopkins PN Toth PP Ballantyne CM Rader DJ Robinson JG. Familial hypercholesterolemia: screening, diagnosis and management of pediatric and adult patients: clinical guidance from the National Lipid Association Expert Panel on Familial Hypercholesterolemia. J Clin Lipidol. (2011) 5:S1–8. 10.1016/j.jacl.2011.03.001 21600525

[43] Kamar A Khalil A Nemer G. The digenic causality in familial hypercholesterolemia: revising the genotype-phenotype correlations of the disease. Front Genet. (2021) 11:572045. 10.3389/fgene.2020.572045 33519890 PMC7844333

[44] Fahed AC Khalaf R Salloum R Andary RR Safa R El-Rassy I. Variable expressivity and co-occurrence of LDLR and LDLRAP1 mutations in familial hypercholesterolemia: failure of the dominant and recessive dichotomy. Mol Genet Genomic Med. (2016) 4:283–91. 10.1002/mgg3.203 27247956 PMC4867562

[45] Besseling J Kindt I Hof M Kastelein JJ Hutten BA Hovingh GK. Severe heterozygous familial hypercholesterolemia and risk for cardiovascular disease: a study of a cohort of 14,000 mutation carriers. Atherosclerosis. (2014) 233:219–23. 10.1016/j.atherosclerosis.2013.12.020 24529147

[46] Cohen H Stefanutti C Di Giacomo S Morozzi C Widhalm K Bjelakovic BB. (2021). Current approach to the diagnosis and treatment of heterozygote and homozygous FH children and adolescents. Curr Atheroscl Rep. 23:30. 10.1007/s11883-021-00926-3 33963467 PMC8105241

[47] Goldstein JL Brown MS. Molecular medicine. The cholesterol quartet. Science. (2001) 292:1310–2. 10.1126/science.1061815 11360986

[48] Dedoussis GV Skoumas J Pitsavos C Choumerianou DM Genschel J Schmidt H. FH clinical phenotype in Greek patients with LDL-R defective vs. negative mutations. Eur J Clin Invest. (2004) 34:402–9. 10.1111/j.1365-2362.2004.01351.x 15200491

[49] Weiss N Binder G Keller C. Mutations in the low-density-lipoprotein receptor gene in German patients with familial hypercholesterolaemia. J Inherit Metab Dis. (2000) 23:778–90. 10.1023/A:1026704517598 11196104

[50] Jiang L Sun LY Dai YF Yang SW Zhang F Wang LY. The distribution and characteristics of LDL receptor mutations in China: a systematic review. Sci Rep. (2015) 5:17272. 10.1038/srep17272 26608663 PMC4660303

[51] Rudenko G Deisenhofer J. The low-density lipoprotein receptor: ligands, debates and lore. Curr Opin Struct Biol. (2003) 13:683–9. 10.1016/j.sbi.2003.10.001 14675545

[52] Holla ØL Kulseth MA Berge KE Leren TP Ranheim T. Nonsense-mediated decay of human LDL receptor mRNA. Scand J Clin Lab Invest. (2009) 69:409–17. 10.1080/00365510802707163 19148831

[53] Li Y Lu W Schwartz AL Bu G. Degradation of the LDL receptor class 2 mutants is mediated by a proteasome-dependent pathway. J Lipid Res. (2004) 45:1084–91. 10.1194/jlr.M300482-JLR200 14993243

[54] Kurosaki T Maquat LE. Nonsense-mediated mRNA decay in humans at a glance. J Cell Sci. (2016) 129:461–7. 10.1242/jcs.181008 26787741 PMC4760306

[55] Kamal NM Sahly AN Banaganapalli B Rashidi OM Shetty PJ Al-Aama JY. Whole exome sequencing identifies rare biallelic ALMS1 missense and stop gain mutations in familial Alström syndrome patients. Saudi J Biol Sci. (2020) 27:271–8. 10.1016/j.sjbs.2019.09.006 31889847 PMC6933154

[56] Galicia-Garcia U Benito-Vicente A Uribe KB Jebari S Larrea-Sebal A Alonso-Estrada R. Mutation type classification and pathogenicity assignment of sixteen missense variants located in the EGF-precursor homology domain of the LDLR. Sci Rep. (2020) 10:1727. 10.1038/s41598-020-58734-9 32015373 PMC6997160

[57] Kirimtay K Temizci B Gültekin M Yapici Z Karabay A. Novel mutations in ATP13A2 associated with mixed neurological presentations and iron toxicity due to nonsense-mediated decay. Brain Res. (2020) 1750:147167. 10.1016/j.brainres.2020.147167 33091395

[58] Pawlicka K Kalathiya U Alfaro J. Nonsense-mediated mRNA decay: pathologies and the potential for novel therapeutics. Cancers (Basel). (2020) 12:765. 10.3390/cancers12030765 32213869 PMC7140085

[59] Hegele RA Tsimikas S. Lipid-lowering agents. Circ Res. (2019) 124:386–404. 10.1161/CIRCRESAHA.118.313171 30702996

